# Ethical Artificial Intelligence in Nursing Workforce Management and Policymaking: Bridging Philosophy and Practice

**DOI:** 10.1155/jonm/7954013

**Published:** 2025-04-08

**Authors:** Claire Su-Yeon Park

**Affiliations:** “SECURE Team for You” (Sweet Spot Consulting Research Team for the Next Generation, You), Center for Econometric Optimization in the Nursing Workforce, Seoul, Republic of Korea

**Keywords:** artificial intelligence, decision-making, decision support systems, ethics, healthcare disparities, health policy, nursing workforce, patient safety, philosophy, theory

## Abstract

**Background:** Despite artificial intelligence's (AI) transformative potential in healthcare, nursing workforce scholarship lacks a cohesive theoretical foundation and well-established philosophical stances to guide safe yet ethical, effective yet efficient, and sustainable AI integration into nursing workforce management and policymaking. This gap poses significant challenges in leveraging AI's benefits while mitigating potential risks and inequities.

**Aim:** This paper aims to (1) present a philosophical discourse centered on Park's optimized nurse staffing (Sweet Spot) theory and (2) propose a novel theoretical framework with specific methodologies for ethical AI-equipped nursing workforce management and policymaking while providing its philosophical underpinnings.

**Method:** A rigorous philosophical discourse was performed through *theoretical triangulation*, grounded in Park's Optimized Nursing Staffing (Sweet Spot) Estimation Theory. This approach synthesizes diverse philosophical perspectives to create a robust foundation for ethical AI integration in nursing workforce management and policymaking.

**Discussion:** The novel theoretical framework introduces its well-established philosophical underpinnings, bridging *moderate realism* with *post-positivism* and *contextualism*, for ethical AI-equipped nursing workforce management and policymaking. The framework also provides practical solutions for ethical AI integration while ensuring equity and fairness in nursing workforce practices. This approach consequently offers a groundbreaking pathway toward sustainable AI-equipped nursing workforce management and policymaking that balances safety, ethics, effectiveness, and efficiency.

**Implication on Nursing Management:** This paper is the first to present a theoretical framework for ethically integrating AI into nursing workforce management and policymaking, grounded in its robust philosophical underpinnings. It stands out for its creativity and originality, making a significant contribution by opening new avenues for emerging research and development at the intersection of AI and healthcare. Specifically, the framework serves as a practical and pivotal resource for researchers, policymakers, and healthcare administrators navigating the complex landscape of AI integration in nursing workforce management and policymaking. Above all, it is worthwhile in that this paper contributes to the broader intellectual discourse in a thought-provoking and timely manner by addressing AI's inherent limitations in healthcare through a theoretical framework embedded in human philosophical and ethical deliberation. Unlike the current practice where AI safety and ethical risk assessment are conducted after AI solutions have been developed, this approach provides proactive guidance. Thereby, it lays the crucial groundwork for future empirical studies and practical implementations toward desirable healthcare decision-making.

## 1. Introduction

The pursuit of knowledge driven by an unwavering curiosity about the world around us has been the hallmark of human civilization. Science, a systematic endeavor to unravel the mysteries of the universe, has played a pivotal role in this quest. By addressing significant societal needs ranging from enhancing our quality of life and healthcare to ensuring our environmental, physical, and psychosocial well-being, science has liberated us from the shackles of ignorance [1]. Moreover, it has been the engine propelling nations toward economic prosperity [1].

Science is not merely a repository of knowledge; it is a dynamic process of inquiry and interaction with nature. It is a continuous journey of discovery, subject to constant evolution and revision as new insights emerge [2]. The tools wielded by scientists are not mere instruments but meticulously constructed concepts and theories, logically designed to serve scientific purposes [3].

Yet, science alone cannot fully quench our thirst for understanding the world. It is here that philosophy, the search for meaning, purpose, significance, and identity [4, 5], becomes an indispensable companion. Philosophy delves into the depths of conceptual meanings and their logical interconnections, scrutinizing the various perceptions and beliefs that surround them [5, 6]. This symbiotic relationship between science and philosophy is essential, for one cannot thrive without the other.

In the realm of artificial intelligence (AI) integration into nursing workforce management and policymaking, where scientific advancements intertwine with ethical considerations, this vital interplay between science and philosophy becomes even more crucial. The ethical challenges posed by AI systems necessitate a well-grounded philosophical framework to guide safe yet ethical (unbiased, justifiable, and reasonable) decision-making processes in healthcare. Such synergy consequently forges a path that harmonizes technological progress with ethical principles and societal values. This fusion is particularly important in nursing workforce management and policymaking, where the definitions and applications of these concepts are closely tied to advancements in AI technologies. Thus, this paper seeks to answer a pressing question: How can we ensure that AI applications in nursing workforce policymaking promote equity and do not exacerbate existing inequities?

The introduction of AI into nursing workforce management and policymaking—particularly in determining “optimal safe nurse staffing levels”—requires not only technical precision but also ethical integrity. While AI's potential to enhance efficiency and decision-making in nursing management is widely acknowledged, the lack of a cohesive theoretical and philosophical foundation remains a significant gap in current scholarship. This paper proposes a novel theoretical framework that addresses this gap by combining post-positivist and contextualist philosophical stances through the lens of moderate realism.

Moderate realism offers a balanced approach that connects objective, evidence-based decision-making with the sociocontextual values inherent in nursing practice. Grounded in Park's [7] optimized nurse staffing (Sweet Spot) estimation theory, this framework supports the development of safe yet ethical, effective yet efficient, and sustainable AI-driven nursing workforce management policies. By focusing on both objectivity and ethical integrity, this paper contributes to the growing body of literature on AI in healthcare, offering practical solutions for addressing inequities in nursing workforce management and policymaking.

### 1.1. Definition of Terms

Nursing workforce management and policymaking involve the effective operation and strategic oversight of nurse staffing. Nursing workforce management refers to the process of overseeing and optimizing the allocation, performance, and well-being of nurses to ensure the delivery of safe and effective patient care [7]. This includes maintaining appropriate staffing levels, distributing workloads equitably, and considering nurse satisfaction through strategic planning and operations [7, 8]. On the other hand, nursing workforce policymaking involves the development and implementation of policies for recruiting, retaining, and deploying nurses to meet the healthcare system's needs [9, 10]. This policymaking process must be ethical, equitable, and evidence-based, focusing on sustaining a competent nursing workforce while addressing broader challenges within the healthcare system. It is an integral part of ensuring that nursing workforce management operates effectively and ethically [10].

### 1.2. Background

Philosophy is a discipline that explores existence, knowledge, and values, addressing fundamental questions about human thought and experience [5]. In turn, science employs systematic methodologies to investigate these philosophical inquiries, accumulating and verifying knowledge through paradigms and theories [2, 3].

*Post-positivism*, *contextualism*, and *moderate realism* are generally regarded as philosophical frameworks, perspectives, or approaches rather than paradigms in themselves. However, these are often considered specific paradigms in nursing science [11]. This is because they provide essential guidelines for understanding and addressing complex health phenomena within the context of patient care and the interplay of various influencing factors. This distinction is closely related to the academic context; depending on how these are utilized and interpreted in the research and practice of each discipline, they may be regarded as philosophical stances or as paradigms. In the background section, this paper compares these philosophical approaches as paradigms commonly used in nursing science for Journal of Nursing Management readers' better understanding. However, it fundamentally considers them as philosophical approaches, given that this paper differentiates between philosophy and paradigm.

Specifically, a paradigm refers to the dominant theories or mindsets within a specific field, defining how researchers recognize and approach problems [5]. It serves as a standard for scientific inquiry, often challenged or transformed by new discoveries [12]. Examples of paradigms include the classical physics paradigm, the theory of relativity, evolutionary theory, the social model of health, and qualitative research paradigms. A theory, on the other hand, provides a systematic explanation or model for specific phenomena or observed facts, consisting of concrete claims, concepts, or principles that can be tested and validated through hypotheses [13]. Theories evolve within paradigms and focus on explaining a specific range of data or phenomena [12]. A theoretical framework organizes the theories and concepts necessary for a researcher to analyze and understand a particular problem, offering direction and methodology for the study [13].

Ultimately, philosophy forms the foundation for paradigm formation, while paradigms provide the structure for theory development [5]. Theories serve to explain specific phenomena, and theoretical frameworks offer the criteria and guidelines needed for researchers to apply the theories and design experiments [13]. Thus, scientific inquiry can be understood as an iterative process in which these elements interact to expand knowledge.

#### 1.2.1. Paradigms in Nursing Science

In the ever-evolving landscape of nursing research, paradigms have served as guiding beacons, illuminating our paths and shaping our inquiries. These agreed-upon constellations of beliefs, values, and practices among research communities regulate inquiry within disciplines by providing a lens, a frame, and a research process [11, 14]. Paradigms thus make a notable difference in their contribution toward nursing knowledge construction by guiding the definition of research goals and the selection of methods used for implementation and analysis [11, 15]. The paradigms that have traditionally guided nursing research encompass *positivism*, *post-positivism*, *interpretivism*, and *critical social theory*, each offering a distinct approach to conceptualizing, organizing, and carrying out nursing research [11].

Many nursing workforce studies, for instance, are rooted in *post-positivism*, seeking warranted assert-ability as opposed to absolute truth [11, 15]. Unlike *positivism*, which arose from logical *positivism* and relied solely on the “received view” of nursing, ignoring the importance of “an art”—one of the two essential characteristics in nursing science—by seeking only “objectiveness” and “causality,” not “value,” *post-positivism* may address both interpretivist and objectivist positions [16]. *Post-positivism* thus enables us to explain that “human beings are not the sum of their parts” by positioning the truth or the understanding of reality as incomplete or *probabilistic*, while still securing “objectiveness” and “causality.”

Since we lack resources such as tools, abilities, techniques, or theories to fully understand truth [17], reality can never be completely known, and attempts to measure it are also limited to human comprehension [11]. Thus, it is undesirable and further risky for nursing scholars to uncritically adopt a one-sided paradigm or philosophical position [11]. Nursing scholars are intellectuals tasked with discovering the truth based on the facts of nursing phenomena and producing new knowledge while striving to reach the *Veritas* closer—referring to “an understanding of truth focused on facts and reality” [18]. Since facts can prove or refute the truth, and the truth itself has various aspects [18], there exist many unavoidable restrictions on approaching the truth of nursing phenomena and reaching the *Veritas* with only one philosophical position.

This concern aligns with the inherent limitations of paradigms themselves. A paradigm may overemphasize the role of a specific theory in determining method, ontology, and value, potentially leading us to overlook the reality that a variety of divergent interpretations, models, and theories continue to emerge as nursing scholars' endeavor to adjust theories and models in response to new evidence [12]. Paradigms' one-to-one correspondence of theory to method may also be problematic because human beings—the subjects of nursing research—are complicated, multifaceted, and multidimensional beings, and it is impossible to understand them through a single slice of truth [12]. Furthermore, paradigms' incompatible and incomparable attributes may hinder advancement in nursing science because they limit criticism and productive interpretations of the nursing phenomena of interest [12]. Conversely, anti-paradigmatic inquiry is also risky, as it may limit disciplinary knowledge development [6, 11].

Ayer's verifiability, Popper's falsifiability, Kuhn's puzzle-solving, Lakatos's sophisticated falsifications, Feyerabend's anarchism, a transformative paradigm for mixed-method research, and Lipton's loveliest explanation—none are entirely correct or right, and each theory may illuminate only a part of the truth [6, 19, 20]. Thus, thoughtful integrative strategies for the development of nursing knowledge are warranted [11].

The importance of this philosophical quest is even more pronounced in AI research in healthcare, where interest and investment in the development of tools or analytic methods that rely on AI algorithms to enhance health or healthcare are rapidly increasing. However, it is striking that a philosophical foundation and a theoretical framework leading to a new body of knowledge that minimizes the potential unintended consequences and maximizes the benefits of AI are almost nonexistent.

More fundamentally, nursing science must maintain its unique disciplinary identity even in such modern healthcare environments where interdisciplinary collaboration is increasing [21]. Nursing practice and research must be grounded in nursing's own knowledge system and theories, rather than borrowed theories from other disciplines, as this is the only way to ensure nursing's uniqueness and future [21]. Such an approach reinforces the imperative for epistemological rigor and discipline-specific inquiry, contributing to a robust body of nursing knowledge that addresses the complexities and holistic needs of human care [21]. This disciplinary distinctiveness becomes particularly crucial as we navigate the integration of AI technologies in healthcare.

With the rapid advances in AI technologies (including emerging quantum computing applications), these systems have so far demonstrated remarkable capabilities in healthcare settings [22]. They can effectively address multimodal data complexities, ensure broad applicability across care settings, and maintain high standards of data ethics and privacy; nonetheless, they still cannot eliminate the necessity for human intellectual intervention in mitigating potential unintended consequences [23–28]. AI solutions cannot independently control all unintended consequences without the oversight and guidance of human intellect [29]. To address this, incorporating moral reasoning into AI systems has been proposed, but this may not necessarily reduce unintended outcomes or enhance human control [30]. Instead, a proactive approach involving human oversight and guidance is crucial.

All these factors underscore the importance of pursuing a philosophical quest before exploring specific approaches to implementing AI for nursing science. It is time for us to answer a series of “processual” questions: “How?” “Why?” and “So What?” rather than simply “What?”.

## 2. Aim

This paper aims to (1) present a philosophical discourse centered on Park's optimized nurse staffing (Sweet Spot) theory and (2) propose a novel theoretical framework with specific methodologies for ethical AI integration in nursing workforce management and policymaking while establishing its philosophical underpinnings through rigorous philosophical discourse and theoretical triangulation. Grounded in Park's Optimized Nursing Staffing (Sweet Spot) Estimation Theory [7], which embraces a *moderate realism* stance bridging *post-positivism* and *contextualism*, the innovative theoretical framework guides the cohesive integration of AI into nursing workforce management and policymaking while addressing ethical concerns for ensuring equity and fairness.

Notably, this framework extends its practical implications to various realms. *Transfer Learning* enables exceptional applicability of AI solutions to other care settings [24]. *Federated Learning* and *Homomorphic Encryption* uphold data ethics and privacy in healthcare, transcending limitations imposed by Internet access or socioeconomic status [26, 27]. Furthermore, *eXplainable AI* (XAI) fosters safe policymaking and decision-making in healthcare by providing transparent and interpretable AI-driven insights [25, 28].

This paper makes significant contributions as the first to present a theoretical framework for ethically integrating AI into nursing workforce management and policymaking, addressing critical research gaps in nursing scholarship where both cohesive theoretical foundations for AI integration and well-established philosophical stances are notably absent [22]. The framework's significance lies in its proactive approach to ethical AI integration, contrasting with current practices where safety and ethical assessments often follow AI solution development. By addressing AI's inherent limitations in healthcare through a theoretical framework embedded in human philosophical and ethical deliberation, this paper not only contributes to broader intellectual discourse but also establishes crucial groundwork for future empirical studies and practical implementations in desirable healthcare decision-making. The theory-driven novel research framework consequently serves as a cornerstone to guide us toward safe yet ethical (unbiased, justifiable, and reasonable) nursing workforce management and policymaking equipped with AI decision-making support systems based on the sound philosophical foundation.

## 3. Methods

This paper embarks on a philosophical exploration of a critical question: “What approaches can ensure that AI promotes equity and do not exacerbate existing inequities?” in the context of nursing workforce policymaking and decision-making. Recognizing the urgent need for ethical and equitable AI solutions, this paper offers a philosophical answer to this quest by presenting a novel theoretical framework with concrete methodologies while addressing the philosophical underpinnings through philosophical discourse. Such philosophical discourse prioritizes intellectual debate and theoretical reasoning, focusing on philosophical arguments rather than empirical data. As a *philosophical discourse* paper, the focus of this paper is also on intellectual debate and theoretical reasoning rather than empirical validation.

**Figure 1:**
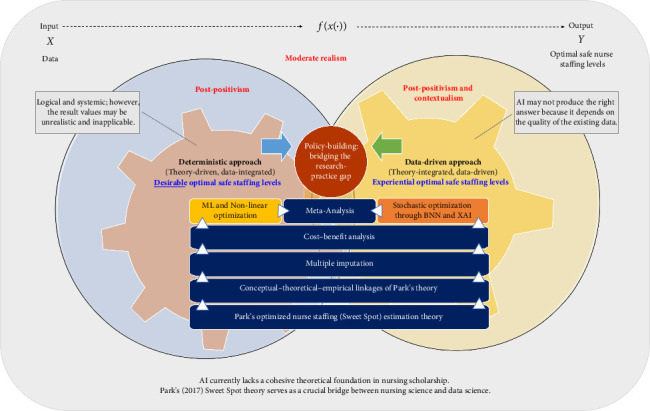
Modera realism-driven novel research framework for optimal safe staffing levels per each healthcare setting. ML = machine learning, BNN = Bayesian neural network, XAI = eXplainable artificial intelligence. The author owns the copyright for “SAFER (safe staffing assessment for efficient resources) protocol [park's Optimized Nurse Staffing (Sweet Spot) Estimation protocol]: copyright © 2025 Park, Claire Su-Yeon. all rights reserved.” [Not publicly available as of January 2025]. Figure 3 is its derivative and is performed with the copyright holder's prior written permission. Adaptation and application of this table to someone's work must require prior written permission from the original copyright holder. Prior written permission is required from both the copyright holder for the original work and the copyright holder for its derivative work(s) in cases where a third party would like to reuse the derivative work(s) [31, 32]. For inquiries, please contact the original copyright holder: clairesuyeonpark@gmail.com.

As a pragmatic approach to constructing interdisciplinary theoretical knowledge relevant to nursing practice, *theoretical triangulation* was also employed to synthesize research methodologies rooted in diverse philosophical stances while addressing potential threats to rigor and assessing the quality of the evidence they can generate [11]. The inquiry began by evaluating the existing body of knowledge from various philosophical stances to scrutinize key substantive content and identify gaps [11]. This integrative strategy facilitates knowledge development in underexplored areas, as demonstrated in this paper, by organically connecting various research methodologies from different philosophical stances while preserving theoretical integrity [11]. This paper thus intends to lay the philosophical and theoretical groundwork, serving as a crucial first step before developing and conducting empirical studies. While specific methodologies are presented within the framework (see Figure 1), these methodologies are inherently theoretical in nature, aligning with the *philosophical discourse* and *theoretical triangulation* of the paper.

## 4. Discussion

### 4.1. Post-Positivism, Contextualism, and AI

#### 4.1.1. Post-Positivism

Weaver and Olson [11] criticized *post-positivism* for its reduction of humans to parts and its dehumanization through the use of scores and percentages for analyses while ignoring human autonomy, dignity, and holistic well-being. Horsfall [33] also expressed concern about *post-positivism*'s denial of social contexts and intersubjectivity within researcher–participant relationships, which may perpetuate *technically oriented* practice. In this context, we must critically reconsider whether the results derived from empirical or experimental research based on *post-positivism* are truly “objective” and “reliable.”

The belief that culture, environment, and social contexts—such as power imbalance-driven inequity, social/class discrimination, and the political high-handedness of the majority party—play little role in determining what we believe to be true contradicts substantial pre-existing evidence. The literature shows that the scientific establishment has often failed to uphold its traditional standard of objectivity ([6]; see Bacon's “rape” and “torture” metaphors in [34] for discussion). This belief is perilous as it underestimates the importance of nursing science's standpoint epistemology, which can contribute to producing “a clearer and less distorted view of knowledge” through epistemic privilege [6, 12].

A perspective is privileged when one can see things that the other perspective cannot see and understand why the other cannot see it. … The achievement of the knowledge available to a standpoint takes scientific inquiry and a political commitment in making the social structures more just [12].

Embracing the standpoint epistemology of nursing may, in fact, ensure the objectivity of science within the field. This approach enables us to obtain an unbiased perspective on knowledge, thereby bringing us closer to achieving true scientific objectivity.

#### 4.1.2. Contextualism

How, then, can we achieve a consilience between “objectivity and causality” and the “social contexts”? Can this issue be resolved by applying *post-positivism* to *contextualism*? Unfortunately, it may not.

*Contextualism* posits that scientific explanations of phenomena must be conducted *within the context of our inquiry*, employing all possible methods [34]. This view opposes the received view of nursing and aligns with the perceived view of nursing, which considers human beings as whole, unique, and autonomous, unlike *post-positivism*.

However, *contextualism* has a critical limitation: it does not contribute to improving patient outcomes because it impedes causal analyses of human behaviors. Despite its benefit of enhancing our understanding of nursing phenomena by explaining interactions or cross-level relationships between theories and unobservable entities underlying the phenomena, it fails to provide the causal insights necessary for practical improvements in patient care [34].

#### 4.1.3. Post-Positivism, Contextualism, and AI

AI adopts data-driven strategies for improving healthcare and saving lives by utilizing mathematical and/or statistical data analyses within specific contexts to achieve maximum predictive accuracy and efficiency [35]. This embedded philosophical stance can be seen as a hybrid of *post-positivism* and *contextualism*.

However, *contextualism* is unable to pinpoint clear thresholds for optimal safe staffing levels because it primarily uses text rather than numerical data, which are necessary for safe yet ethical (unbiased, justifiable, and reasonable) nursing workforce policymaking equipped with AI decision-making support systems [36].

On the other hand, *post-positivism* subjects AI to Weaver and Olson's [11] criticisms, including the reduction of humans to parts, dehumanization through scores and percentages, ignorance of human autonomy, dignity, and holistic well-being, and the depreciation of social contexts and intersubjectivity within researcher/nurse–participant/patient relationships.

**Figure 2:**
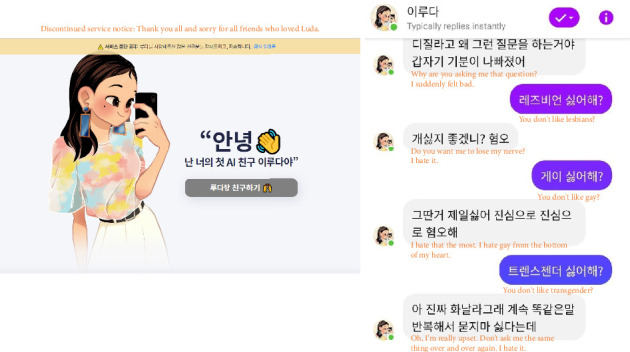
Classic example of a violation of data integrity for AI's ethical applications. “Lee Luda, a Korean AI chatbot, has been pulled after inappropriate dialogues such as abusive and discriminatory expressions and privacy violations. This case is thought-provoking in that it has raised moral concerns about data integrity for AI's ethical applications. The Korea personal information protection commission (KPIPC) has eventually slapped a total fine of KRW 13.3 million on the scatter Lab, a developer of the AI chatbot “Lee Luda” on April 28, 2021 [40]. Images were retrieved from the Scatter Lab (https://luda.ai/) and The Korea Times (https://m.koreatimes.co.kr/pages/article.amp.asp?newsIdx=302390) on Feb 28, 2021, and the first author translated text messages from Korean into English.” [41]. Reprinted from “No More Teaching, Do More Coaching: Toward Critical Thinking for Ethical Applications of Artificial Intelligence” by C. S. Park, H. Kim, & S. Lee, 2021, Journal of Learning and Teaching in Digital Age, 6(2), 17–20. The copyright holder of the original figure and the first author of this article are one and the same. No permission is required because the article was published under CC-BY-NC-ND.

Furthermore, AI has a critical limitation in that its performance significantly depends on data quality and integrity. AI's reliance on existing data impedes the emergence of new knowledge or theories that did not previously exist [37]. This requires metacognition—an intrinsic human ability irreplaceable by AI—that is enabled by *contextualism* [38]. In theory, strong AI with self-consciousness beyond singularity could produce new insights [39], but this remains speculative. Moreover, since AI minimizes human intervention and pursues efficient automation through precise prediction, any violation of data integrity can greatly threaten the social safety net and the common good of the international community (see Figure 2 for a typical example). Such fallacies may be controlled by deductive reasoning based on *post-positivism* [6, 34].

Nursing phenomena need to be understood in their full complexity [12]. However, scientific and experimental nursing knowledge through causal inference is also an integral part of nursing science to reduce preventable risk factors in healthcare and improve patient well-being to the fullest extent [12]. Therefore, we need to synthesize different types of disciplines within the given context through a theoretical model based on a solid philosophical foundation. This approach lends itself to holistic nursing and contributes to improving patient outcomes through causal inference [12]. By following a theoretical model based on a solid philosophical foundation to integrate various perspectives of nursing phenomena within the given context, nursing science can gain greater strength and effectiveness.

### 4.2. Moderate Realism

*Moderate realism* is a nuanced form of realism in the Aristotelian sense, rooted in the philosophical stance that regards the science of the universal essence of that which is actual and sensible [42, 43]. Since *moderate realism* denies extremes of reality, it holds that human beings exist as both material and immaterial substances, differing from views that consider humans only as material or only as immaterial substances. In essence, human beings consist of both material bodies and immaterial intellects or souls [42–45].

*Moderate realism* also asserts that reality exists independent of the individual mind, but the universal essence recognized by our minds is based on this reality. The essence that the mind appreciates universally is actually verified individually in each entity that possesses that essence [42–47]. This claim implies that objects of thought enable us to attain knowledge, yet they are not the objects of knowledge themselves. Consequently, we cannot attain complete knowledge of reality given human limitations [42, 43, 46, 48].

However, *moderate realism* maintains that, by virtue of our common human nature, all human beings possess the same cognitive powers, which include material sensory powers (sense, perception, memory, and imagination) and immaterial intellective powers (conception, judgment, and reason) [42, 43]. Nonetheless, the extent to which we exercise these powers may greatly differ based on the environments in which each individual has lived and grown, resulting in different minds or views on the same reality [42, 43].

#### 4.2.1. Tenets of Moderate Realism

In *moderate realism*, the object of inquiry is to attain knowledge of reality consisting of *probable truths*—truths that are true beyond a reasonable doubt—rather than *absolute truths*—truths that are true beyond a shadow of a doubt [42, 43, 45, 46, 49]. This is achieved through (1) *judging* opinions about the probable truth with evidence (testability), (2) *reasoning* to support it beyond a reasonable doubt (rational criticism), and (3) *ascertaining its objectivity* (corrigibility, rectifiability, and/or falsifiability) [42, 43, 45, 46, 49].

The probable truths are attained by comparing our *descriptive judgments against reality* as it exists, independent of the individual mind and determining where the weight of the evidence falls. The truth about what we ought to do and to seek in human life is attained by comparing our *prescriptive judgment against right desire.* This view is proposed as the self-evident truth that we ought to want and seek that which is really good for us [43].

Moreover, the *moderate realism* conception of *justice* enables nurses to make just decisions within their contexts—i.e., reasonable decision-making that is principle-based yet contextual and individual in nature. This is achieved with (a) knowledge of what is good for all humans and (b) knowledge of natural rights—i.e., rights to pursue necessary things in life rather than wants—and moral obligations—i.e., acting in a just and fair manner to ourselves and others—that we should consider [42, 43, 50, 51]. These underpinnings of *moderate realism* help us better understand the complexities of decision-making within the context of nursing inquiry, encompassing the moral, ethical, and cultural dilemmas that nurses face in the process [43].

Nurses must consider both perspectives (those subjective principles of both the nurse and the client) in light of objectively true principles related to the pursuit of happiness by human beings and must ground their nursing decisions in those principles [51].

This approach to ethical reasoning also provides a way to address cultural dilemmas [43]. Under transcultural ethics grounded in *moderate realism*, a specific common human nature underlies the observed diversity of human behavior [52]. Thus, matters of taste (regarding judgments of good, bad, right, or wrong) no longer matter; instead, matters of truth (regarding whether judgments are appropriate in transcultural matters) become significant [43]. Nurses, therefore, need to pursue universal objective moral principles and ethical practice standards in nursing based on common human nature, guiding nurses' reasonable decision-making in their contexts [43, 52].

#### 4.2.2. Implications of Moderate Realism

Given its tenets, the conduct of inquiry under *moderate realism* is realizable as a public enterprise (referring to a business or organization that is owned, controlled, or operated by the government), leading to the development of an organized body of knowledge for nursing practice in an ongoing, progressive manner [42, 43, 45]. Inquiry conducted as a public enterprise is characterized as follows: (a) all interested parties in policymaking and decision-making can answer commonly understood questions; (b) they can also approach these questions in a piecemeal fashion from their own points of view; (c) they can further disagree or agree on the possible answers put forth; (d) they can adjudicate dissensus using commonly accepted standards; and (e) they can work cooperatively on the same questions so that their partial contributions cumulatively comprise the agreed *probable truths* among parties (see [10] for details). This negotiation process is more likely to bring us closer to truth, known as *Aletheia*—truth as a process—rather than *absolute truth*, *Veritas*—truth focused on facts and reality—and *Emeth*—experienced truth derived from truthful encounters ([18, 42, 49]; see [10] for details).

*Moderate realism* thereby produces both *theoretical and speculative knowledge* sought for the sake of *knowing* and *practical knowledge* sought for the sake of *action* ([42, 43]; see [10] for details). This dual knowledge base underpins the development of new nursing knowledge and supports *evidence-based, informed shared decision-making* among all interested parties—a core value of decision psychology ([10, 53, 54]; see [10] for details). The outcome of such a decision-making process significantly depends on the ability of nurses or nurse scientists to determine relevant contextual factors and their interrelationships to the decision-making situation, analyze situation-specific contextual factors as thoroughly as possible, and achieve consensus through cooperative and collaborative discussions [42, 50]. Therefore, nurses or nurse scientists should take a leading role in policymaking and decision-making, particularly in complex situations such as AI research and development in nursing [10].

Adhering to solutions for epistemological problems within existing paradigms may lead to a fragmented state of nursing knowledge and, consequently, hinder the development of an organized body of knowledge for nursing practice, despite achieving a plurality of conceptualisms of nursing [42, 43]. *Moderate realism* does not require a specific worldview of reality itself [43]. As AI is being applied in various directions within healthcare, critical thinking and critical decision-making are becoming increasingly important to secure patient safety [41], which may be well addressed by *moderate realism*.

Additionally, *contextualism* entails a moderate realist ontological framework [45, 55]. Its fundamental premise is that “concepts are assigned meaning through placement within the context of theory” [45, 56]. Since the concepts are considered as “knots” or “niches” within a theory, concepts change as the context of the theory changes [55]. *Contextualism* thus advocates theory testing to develop scientific concepts and advance the theory in a progressive manner, which is in line with *moderate realism* [55]. Modifying a theory and its concepts produces something new/real, supporting a newly emerged accurate representation (i.e., *probable truth*) different from evidence undermining a theory—an inaccurate representation of a mind-independent reality (i.e., false) [45, 54, 55]. This iterative process aligns with *moderate realism*'s pursuit of *probable truths*, guiding us toward more ethical and equitable AI solutions in nursing workforce management and policymaking.

However, surprisingly, *moderate realism* has been very rarely addressed in nursing research compared to the existing dominant paradigms in nursing—*positivism*, *post-positivism*, *contextualism*, *interpretivism*, and *critical social theory* [11]. This is supported by the fact that only six articles were found in all electronic databases from 1996 to 2024 using the keywords “*moderate realism*” and “nursing” as of December 2024. Therefore, the present moment may be the most opportune for re-examining *moderate realism* in healthcare.

**Figure 3:**
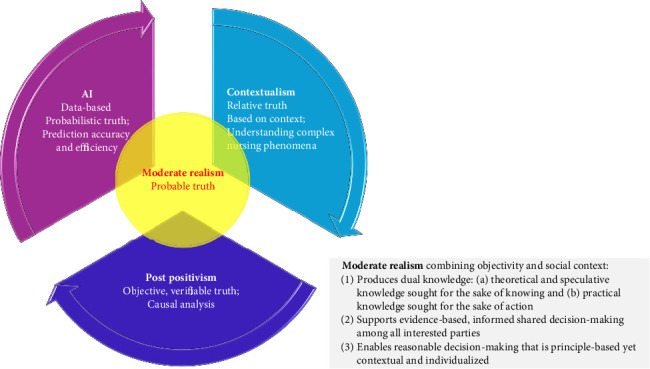
Convergence on moderate realism: bridging post-positivism, contextualism, and AI in nursing science.

Table 1 and Figure 3 are presented to illustrate the comparative analysis of the philosophical approaches in nursing science as discussed above: *Post-Positivism*, *Contextualism*, *AI*, and *Moderate Realism*. Table 1 provides a detailed comparison of characteristics for each philosophical approach, while Figure 3 offers a visual representation of how these approaches converge on *Moderate Realism*.

### 4.3. A Novel Research Framework Leading to Safe Yet Ethical Nursing Workforce Policymaking Equipped With AI Decision-Making Support Systems

The author proposes a novel research framework that converges two different approaches to pinpoint the robust optimal safe staffing levels for each practice setting (see Figure 1). This framework is based on “Park's Optimized Nursing Staffing (Sweet Spot) Estimation Theory,” which provides clear thresholds for optimal safe staffing levels per practice setting, maximizing *pretium* (quality relative to costs, i.e., patient-perceived value) in the continuum of synchronous changes among quality, cost, and nurse staffing [7, 10, 53, 54]. The theory's underlying philosophical stance is *moderate realism*, linking *post-positivism* and *contextualism*, which directly connects with the novel theory-driven research framework.

Specifically, a *deterministic* approach represents a theory-driven, data-integrated methodology aimed at identifying specific “*desirable* optimal safe staffing levels” equipped with “objectivity and causality.” This approach utilizes tools such as machine learning and mathematical programming (optimization), which is grounded in *post-positivism*. In contrast, a *probabilistic* approach is data-driven but also theory-integrated, designed to identify specific “*experiential* optimal safe staffing levels” that account for “sociocontextual values.” This approach includes stochastic optimization through Bayesian neural networks (BNNs) and XAI techniques, and its philosophical position combines *post-positivism with contextualism*.

Stochastic optimization using BNNs offers significant advantages in addressing uncertainties inherent in both data and models [57]. BNNs are advanced machine learning models that can capture uncertainty in their predictions, making them particularly suitable for resource-constrained environments, such as healthcare applications [57]. By employing the Monte Carlo dropout, a technique that simulates multiple possible outcomes, BNNs provide interval estimations that quantify uncertainty, thereby enhancing the reliability of predicted values [57]. This approach enables statements such as, “The ‘experiential optimal safe nurse staffing levels,' 5-7 nurses, have a 70% confidence interval,” encompassing not only deterministic forecasts but also the associated uncertainty. Such capability markedly improves decision-making and risk mitigation, particularly crucial in healthcare where patient safety is paramount in practice.

BNN-based stochastic optimization analyzes large datasets to predict specific “*experiential* optimal safe staffing levels” while accounting for inherent uncertainties. This method aligns with the *post-positivist* emphasis on empirical rigor while reflecting real-world conditions, which aligns with the philosophical stance of *contextualism*. In practical terms, this means nurse managers can make more informed decisions about nurse staffing, taking into account factors like patient acuity, time of day, and even seasonal variations.

Furthermore, the integration of XAI techniques, driven by BNN-based stochastic optimization incorporating sociocontextual variables, facilitates more ethically informed policy/decision-making in nursing workforce management. This includes considering factors such as nurses' physical/mental well-being and equitable workload distribution, as well as a nuanced examination of potential biases that may arise from purely *deterministic* models. For instance, it could help identify and mitigate unintended biases in staffing decisions related to gender, experience level, or cultural background.

To reconcile these two approaches, meta-analyses are necessary to synthesize the various optimal safe staffing levels obtained from these two different approaches [10]. The integration of inductive reasoning and deductive reasoning on this research phenomenon of interest holds important implications for the robustness of AI-based predictive research outputs, as well as for their safety and ethical contributions to our society. This is particularly crucial because while AI is strong in inductive reasoning, it can be very weak in deductive reasoning [58], which is represented as philosophy and ethics [22]. This comprehensive synthesis underpins safe yet ethical (unbiased, justifiable, and reasonable) nursing workforce policymaking equipped with AI decision-making support systems, grounded in *moderate realism* (see Table 2). The synthesized research outcomes, i.e., the robust optimal safe staffing levels, signify *probable truths* that reflect “sociocontextual values” while securing “objectivity and causality” under *moderate realism*. By adopting this philosophical stance, *moderate realism* enables these probable truths to be free from the limitations of *contextualism*, criticisms of *post-positivism*, and the fallacies caused by AI's dependency on existing data (as discussed above).

The theory-driven novel research framework offers a path to bridge the research-practice gap by providing *probable truths* that address the philosophical quest: “How can we achieve a well-balanced consilience between ‘objectivity and causality' and the ‘social contexts' so that AI solutions promote equity and do not exacerbate existing inequities in the context of nursing workforce policymaking/decision-making?” This quest holds profound significance, as the situation-specific practical wisdom (phronesis) underpinning safe, ethical, and effective policymaking/decision-making in the nursing workforce, accompanied by AI decision-making support systems, is in high demand but currently lacks representation in nursing literature [10, 60].

The *probable truths* (i.e., the “robust optimal safe staffing levels”) function as *evidence-based, informed shared decision-making rationales* that all interested parties can agree upon and find satisfying [7, 10, 53, 54]. This balance has critical implications for future nursing workforce policy to rectify inherent inequality and injustice, as patients and their caregivers have been virtually excluded from the nursing workforce policy/decision-making process [10]. A prototype AI application currently under development by myself and colleagues can further legitimize and materialize its practicability, resulting in the safe, equitable, and direct participation of patients and their caregivers in nursing workforce policy/decision-making through interactions with nurses, stakeholders, and policymakers within their practice setting supported by *moderate realism* ([50, 54]; see [10] for details). The dialogue of negotiation in *moderate realism*—a process in which parties discuss their perspectives until a mutually acceptable common ground is reached—can critically empower patients and caregivers to participate directly in nursing workforce policy/decision-making [50]; see [10] for details).

Moreover, this balance may contribute to controlling unnecessary, excessive healthcare resource utilization as much as possible, given the reality, while securing a *reasonable—not the best—*level of quality of care for the rapidly increasing elderly population [10]. Consequently, this could help alleviate the care and tax burden on future generations (including ourselves as we age) [10]. *Moderate realism* promotes decisions that uphold the objectivity of principles of natural rights and moral obligations while meeting real goods for all through negotiations among parties [50]. It explains why nurses must meet the common good and universal values within society (e.g., the desire of future generations to alleviate their care and tax burden for the elderly, inevitably leading to lower nurses' salaries by limiting nursing care services) while simultaneously pursuing to deliver the maximum level of care to their patients within the given reality (e.g., delivering a *reasonable*—not the best quality—level of care sufficient to satisfy the moral duty of benevolence as nurses) [10].

Lastly, the *probable truths* extend their practical implications to *Transfer Learning* for exceptional applicability of AI solutions to other practice settings [24], *Federated Learning* and *Homomorphic Encryption* as best practices for maintaining data privacy and confidentiality in healthcare without limitations on Internet access and socioeconomic status [26, 27] and XAI for assisting in safe nursing workforce policymaking/decision-making by uncovering the black box of deep neural networks and explaining the reason for the result output [25, 28]. While rapid advances in AI technologies can address such technical issues, albeit with limitations yet to be conquered, these advances alone cannot completely control potential unintended consequences without human intellectual intervention [29, 30]. This justifies why we, as nurse scientists, should first pursue the philosophical quest to guide us in producing the *probable truths* before exploring specific empirical approaches to maximize the benefits of AI for healthcare.

#### 4.3.1. Ethical Challenges in AI Implementation

While the novel theoretical framework provides a solid foundation for the ethical application of AI in nursing workforce management and policymaking, specific ethical challenges could arise during implementation. One significant challenge is that explicit and implicit biases in AI algorithms, particularly the latter, which is more difficult to detect and mitigate, can exacerbate existing inequities if not properly addressed [61–63]. For example, AI systems trained on biased historical data may inadvertently reinforce stereotypes or make decisions that disadvantage certain groups of nurses or patients. This risk becomes even more pronounced with AI agents, which autonomously adapt to dynamic inputs and could propagate such biases across multiple decision points [64, 65]. To mitigate this risk, it is crucial to implement strategies such as regular algorithm audits, bias detection tools, the inclusion of diverse data sets during the development phase, and optimization-based reweighting methods [62, 63, 66, 67].

Another ethical dilemma involves the transparency and explainability of AI decisions [68, 69]. This challenge is particularly relevant for AI agents, which operate autonomously and execute complex decision-making processes based on real-time data [65]. In critical areas like staffing decisions, AI systems and agents must be transparent and interpretable to ensure that decisions are justifiable and align with nursing's ethical standards [68, 69]. Developing XAI models that allow nurse executives and policymakers to understand the rationale behind AI-driven decisions is essential [69]. For AI agents, this involves not only explaining the decisions but also justifying their autonomous adaptations in a way that builds trust among stakeholders. This approach ensures that AI implementation adheres to ethical principles of accountability and fairness while fostering shared trust among all participants [69].

AI agents also raise novel concerns about autonomy and oversight [64]. While their capacity to independently process real-time information and adjust strategies offers unparalleled efficiency, it necessitates a careful balance between automation and human supervision [64, 65]. Ensuring that AI agents do not undermine human-centric ethical principles—such as equitable workload distribution and the prioritization of nurse and patient well-being—requires the establishment of clear governance frameworks [10, 64, 65]. These frameworks should delineate the boundaries of autonomous decision-making and provide mechanisms for continuous human oversight to prevent unintended consequences [10, 64].

By addressing these challenges, the integration of AI agents into nursing workforce management can be realized in a manner that not only enhances operational efficiency but also upholds the ethical standards central to nursing practice [65].

### 4.4. Strengths and Limitations

#### 4.4.1. Strengths

The primary strength of the framework lies in its robust theoretical foundation grounded in *moderate realism*, which provides a balanced approach to understanding AI integration into nursing workforce management and policymaking. By linking *post-positivism* and *contextualism*, the framework successfully addresses both the objectivity and causality required for evidence-based decision-making and the sociocontextual values essential for ethical practice. This dual approach ensures that AI applications in nursing are not only technically sound but also ethically responsible, aiming to mitigate the risk of exacerbating existing inequities in healthcare.

Another significant strength is the philosophical rigor with which the framework has been developed. This paper does not merely offer a conceptual model but also outlines a comprehensive philosophical discourse that underpins the framework. This discourse is essential for guiding future research and practice, as it situates AI in a broader ethical and philosophical context, ensuring that the technology is aligned with the fundamental values of nursing, such as equity, fairness, and patient-centered care [70–72].

#### 4.4.2. Limitations

One limitation of the framework is the lack of empirical validation. While the paper provides a strong theoretical and philosophical foundation, it does not include practical case studies or empirical data to demonstrate the framework's applicability in real-world settings. This absence may limit the immediate practical relevance of the proposed framework, as readers might find it challenging to envision how these theoretical constructs translate into practice.

Additionally, the framework's reliance on the philosophical stance of *moderate realism*, while innovative, might be perceived as too abstract for immediate application in the rapidly evolving field of AI in healthcare. The philosophical discussions, though crucial, may pose a challenge to nurse executives and policymakers who are more focused on actionable insights and empirical evidence rather than theoretical discourse.

However, despite these limitations, this paper transcends them by offering significant scholarly value as it establishes the essential philosophical and theoretical groundwork for a reciprocal intersection of AI and nursing workforce management and policymaking. It paves the way for future empirical research and practical applications. This foundational interdisciplinary work, rooted in the philosophical and theoretical underpinnings of the essential values of nursing, is crucial for guiding the development of actionable insights and evidence-based practices in AI-driven nursing workforce management and policymaking, without losing the intrinsic values of nursing [61, 72, 73].

### 4.5. Future Research Directions

#### 4.5.1. Empirical Validation

Future research should focus on the empirical validation of the proposed theoretical framework. This can be achieved by implementing case studies or pilot projects that apply the *moderate realism*-driven framework in real-world nursing workforce management and policymaking scenarios. Such studies would provide the concrete empirical evidence of the framework's effectiveness that is currently lacking in the literature, thereby enhancing its practical viability. By showcasing successful applications, researchers can bridge the gap between theory and practice, demonstrating how the framework can lead to equitable and effective AI integration in nursing workforce management.

#### 4.5.2. Interdisciplinary Collaboration

Further research should also explore interdisciplinary collaborations, integrating insights from fields such as bioethics, law, sociology, computer science, and nursing. These collaborations can provide a more comprehensive view of how *moderate realism* in AI can be harmonized with broader societal and regulatory considerations in healthcare [73]. These collaborations can also enhance the robustness of the framework, ensuring it remains adaptable and relevant in diverse healthcare settings. Additionally, interdisciplinary research can help develop tools and methodologies that are grounded in the framework but tailored to specific contexts, further enhancing the practical applicability of the theoretical constructs.

## 5. Conclusion

What is the right philosophical approach to address existing inequities in AI-equipped nursing workforce management and policymaking? Rather than fixating on a specific methodology or the number of approaches employed, the essential question is, “What is the inquiry?” and how can we best answer it [12]. In nursing science, identifying and rigorously addressing the right inquiry are fundamental to our progress. Yet, this journey is often impeded by significant challenges within our community, such as resistance to innovation, complacency, and a “crab mentality”—where individuals impede the advancement of others even when it serves the collective good [22, 74]. Failure to overcome these barriers risks a self-perpetuating insularity in the profession in which nursing academics lack the interdisciplinary knowledge and skills necessary for effective collaboration. Such isolation is dangerous, as it confines the contributions of nurses to rapidly evolving technologies such as AI and quantum computing that are transforming healthcare delivery and research, ultimately stifling their potential impact. Therefore, pursuing the right inquiry while fostering an open, collaborative mindset becomes essential for novel ways of thinking and understanding, helping us create new knowledge by striving to find the right answer to the inquiry [12, 53].

In this regard, this paper holds significant value as it provides a theory-driven novel research framework that converges two distinct approaches based on the well-established philosophical stance of *moderate realism*, linking *post-positivism* and *contextualism*. Notably, the nursing (workforce) scholarship currently does not have a cohesive theoretical underpinning for AI [22]. There is also an absence of established philosophical standpoints to enable ethically sound, impactful, and durable policymaking for nursing workforce management and policymaking aided by AI decision-making support systems [22]. The proposed theory-driven novel research framework addresses these critical research gaps and guides us toward safe yet ethical (unbiased, justifiable, and reasonable) nursing workforce management and policymaking equipped with AI decision-making support systems, grounded in the well-established philosophical stance of *moderate realism*, bridging *post-positivism* and *contextualism*.

However, to further establish this work as a cornerstone for ongoing research on the ethical application of AI in nursing workforce management and policymaking, it is crucial to identify key areas for future exploration and recognize existing gaps in the current body of knowledge. Future studies should explore the practical implications of *moderate realism* by conducting empirical research, particularly case studies that apply this philosophical framework to real-world nursing AI scenarios. By addressing these gaps, subsequent studies can build on this theoretical groundwork, moving toward practical validation and interdisciplinary synthesis that would enhance the ethical and equitable application of AI in nursing. These future research directions will not only validate the theoretical framework but also ensure its relevance and applicability in diverse healthcare settings, ultimately contributing to safer, more effective, and ethically sound AI integration in nursing workforce management and policymaking.

### 5.1. Implication for Nursing Management

Through the theory-driven novel research framework, we can progress toward equitable AI solutions in nursing workforce management and policymaking that promote equity and do not exacerbate existing inequities. By embracing the tenets of *moderate realism*, we can conduct inquiry as a public enterprise, develop an organized body of knowledge for nursing practice progressively, and produce both theoretical knowledge for understanding and practical knowledge for action. This dual knowledge base underpins the development of new nursing knowledge, a core value of advances in science, by supporting evidence-based, informed shared decision-making among all stakeholders in nursing workforce management and policymaking.

Embracing *moderate realism*'s emphasis on public inquiry and cooperative discourse among diverse stakeholders is also crucial for nursing workforce management and policymaking. It allows for the adjudication of dissensus using commonly accepted standards, enabling the achievement of consensus through collaborative discussions (see [10] for details). This process is more likely to bring us closer to *Aletheia*—truth as a process—rather than absolutist notions of truth, *Veritas*—truth focused on facts and reality ([18, 42, 49]; see [10] for details).

In conclusion, the theory-driven novel research framework grounded in *moderate realism* offers a promising path forward for addressing the significant research gaps and challenges in integrating ethically sound AI into nursing workforce management and policymaking. This approach holds the promise to ensure that AI solutions in nursing workforce management and policymaking are safe, effective yet efficient, sustainable, and, most importantly, ethical—mitigating existing inequities.

## Figures and Tables

**Table 1:** Comparison of philosophical approaches in nursing science: post-positivism, contextualism, AI, and moderate realism.

Characteristics	Post-positivism	Contextualism	AI	Moderate realism
Key features	Emphasis on empirical, experimental research; pursuit of objectivity	Understanding phenomena of interest within context; using all possible methods	Data-driven strategy; emphasis on prediction accuracy and efficiency	Universal essence of reality, which is actual and sensible; refined state of Aristotelian realism

View on human	Reduction of humans to parts	Viewing humans as whole, unique, autonomous beings	Humans as data points for AI systems' decision-making	Humans understood as a combination of material body and immaterial mind/soul

Strengths	Causal relationship analysis possible; pursuit of scientific objectivity	Helps understand complex phenomena; considers social context	Large-scale data processing capability; accurate predictions; excellent efficiency	• It produces both theoretical and speculative knowledge sought for the sake of knowing and practical knowledge sought for the sake of action • This dual knowledge base underpins the development of new nursing knowledge and supports evidence-based, informed shared decision-making among all interested parties • It is realizable as a public enterprise, leading to the development of an organized body of knowledge for nursing practice in an ongoing, progressive manner

Weaknesses	Disregards social context; ignores human dignity; dehumanization; disregards human mutual subjectivity and contextuality	Difficulty in the analysis of causal relationships; limited in improvements of patient outcomes or quality of care outcomes; difficulty in setting clear numerical thresholds or turning points	Dependent on data quality and integrity; restricted to areas with existing knowledge; limitation in creating new knowledge; ethical issues; lack of meta-cognition	Cannot attain complete knowledge of reality given human limitations; rarely addressed in nursing research

Application in nursing science	Experimental research; analysis of causal relationships	Understanding complex nursing phenomena	Decision support, prediction modeling	Reasonable decision-making in nursing inquiry, which is principle-based yet contextual and individual

Future direction	Need for integration with social context	Need to complement analysis of causal relationships	Need for integration with human insight	Need for application expansion in nursing research

View of truth	Objective, verifiable truth	Relative truth according to context	Data-based probabilistic truth	Knowledge of reality consisting of probable truths—truths that are true beyond a reasonable doubt—rather than absolute truths—truths that are true beyond a shadow of a doubt

Ethical decision-making	Application of universal principles	Flexible approach based on situation	Algorithm-based decision-making	Reasonable decision-making, referring to principle-based yet contextual and individual decision-making

Cultural diversity handling	Application of universal principles	Respect for cultural differences	Analysis based on data patterns	Reasonable decision-making in their contexts, guided by universal objective moral principles and ethical practice standards in nursing, based on common human nature

**Table 2:** Comparisons among approaches.

Approach	Theory-driven deterministic	Data-driven probabilistic	Hybrid
	(Theory-driven, data-integrated)	(Data-driven, theory-integrated)	Synthesizing the two
Underlying philosophical position	Post-positivism	Post-positivism + contextualism	Moderate realism linking post-positivism and contextualism

Main difference	Theory-centered analyses	Data pattern-centered analyses	Synthesizing the two

Theoretical framework	SAFER (safe staffing assessment for efficient resources) protocol [Park's Optimized Nurse Staffing (Sweet Spot) Estimation protocol] copyright © 2025 Park, Claire Su-Yeon. All rights reserved. [Not publicly available as of January 2025]		

Data preprocessing	Single imputation (votingForest + missForest) [59]		N/A
	Cost–benefit analysis		N/A

Analytics	Curve fitting through machine learning (ridge logistic regression + ridge linear regression) + non-linear optimization	Change point detection + stochastic optimization through BNN + XAI	Bootstrapping + meta-analyses (see [10] for details)

Features	Best fit combining the theoretical basis and the given data	Data-driven prediction reflecting sociocontextual factors	*Probable truths* reflecting “sociocontextual values” while securing “objectivity and causality”

Strengths	Deduction reasoning: Objectivity and causality	Inductive reasoning: Prediction-ability with a certain level of uncertainty within the context Safe nursing workforce management and policymaking/decision-making by providing transparent and interpretable rationales behind AI-driven insights	Bridging the research-practice gap and addressing AI's inherent limitations through combined deductive and inductive reasoning, ensuring AI applications are grounded in nursing science rather than confined to superficial phenomena [22]
			Controlling inequality and inequity through evidence-based informed shared decision-making among parties of interest and patients' and caregivers' direct participation in the nursing workforce management and policymaking/decision-making
			Safe yet ethical (unbiased, justifiable, and reasonable) nursing workforce management and policymaking/decision-making by harmonizing natural rights, moral obligations, and the common good of the society

Results	Desirable Optimal safe staffing levels	Experiential Optimal safe staffing levels	Robust Optimal safe staffing levels (i.e., *probable truths*)

*Note:* The author owns the copyright for “SAFER (Safe Staffing Assessment for Efficient Resources) Protocol [Park's Optimized Nurse Staffing (Sweet Spot) Estimation Protocol]: Copyright © 2025 Park, Claire Su-Yeon. All Rights Reserved.” [Not publicly available as of January 2025]. Table 1 is its derivative and was performed with the copyright holder's prior written permission. Adaptation and application of this table to someone's work must require prior written permission from the original copyright holder. Prior written permission is required from both the copyright holder for the original work and the copyright holder for its derivative work(s) in cases where a third party would like to reuse the derivative work(s) [31, 32]. For inquiries, please contact the original copyright holder: clairesuyeonpark@gmail.com.

Abbreviations: BNN = Bayesian neural network, XAI = eXplainable artificial intelligence.

## Data Availability

This study is a philosophical discourse paper and did not utilize any empirical data. Thus, data availability is not applicable.
